# Evaluation of Junior Doctors’ Experience of Psychodynamic Psychotherapy Training in LYPFT During the COVID-19 Pandemic

**DOI:** 10.1192/bjo.2022.383

**Published:** 2022-06-20

**Authors:** Oliver Turner, Gwenllian Collin, Vikram Luthra

**Affiliations:** 1 Leeds and York Partnership NHS Foundation Trust, Leeds, United Kingdom; 2 Bradford District Care NHS Foundation Trust, Bradford, United Kingdom

## Abstract

**Aims:**

Developing psychotherapeutic competencies is an essential part of psychiatric training. All core trainees in LYPFT until 2021 saw a patient for Psychodynamic Psychotherapy. The pandemic led to unprecedented changes to clinical practice and medical education. In LYPFT all face-to-face appointments in the Medical Psychotherapy Service were paused in March 2020. Patients were offered the choice to continue therapy remotely or postpone therapy. Supervision was also moved to a remote format. Face-to-face psychotherapy sessions resumed from August 2020, with new departmental procedures around infection control and the use of PPE. This project aimed to establish the junior doctors’ experience of delivering psychodynamic psychotherapy in LYPFT during the COVID-19 pandemic.

**Methods:**

The project was carried out via a two-step methodology: Firstly with an online survey which included a quantitative analysis of the impact of the pandemic; and secondly via semi-structured interviews with a resultant thematic analysis.

**Results:**

22 junior doctors who were invited to participate, 15 completing the survey (68%). Four patients had deferred therapy; the mean length of deferral was 2 months. Ten respondents had sessions cancelled due to infection or self-isolation. Face-to-face delivery was experienced by 13 respondents, 5 respondents had delivered therapy via phone and 6 had delivered therapy with PPE. Thirteen were concerned about attaining their psychotherapy competencies. Seven preferred face-to-face supervision, and 4 preferred remote working.

Thematic Analysis of the semi-structured interviews identified three themes regarding the impact of the COVID-19 pandemic on Junior Doctors experience of Psychodynamic Psychotherapy, with sub-themes detailed below. Throughout the themes, the challenges and difficulties with delivering therapy in the COVID-19 pandemic, as well as areas of good practice and opportunities were identified.

The Work of Therapy (Remote Therapy, PPE and Therapy, COVID-19-related)

1. The Structure of Therapy (COVID-19 Guidance, Setting/Frame of Therapy, Boundaries of Therapy)
2. The Therapist's Training (Supervision, Attaining Competencies, Loss of Training Experience)

**Conclusion:**

Recommendations:

1. To create a short guide for junior doctors delivering Psychodynamic Psychotherapy during a pandemic.
2. To consider the types of supervision delivery within the Medical Psychotherapy Service
3. To ensure there is space for junior doctors within the Medical Psychotherapy department or a private space within their base placement, should remote therapy be required.
4. To ensure future plans related to possible pandemic restrictions address the need for good quality and strong internet connections/WIFI

